# Climate-Driven Changes in Potential Suitable Habitat of *Protegira songi* in China Under Future Climate Scenarios

**DOI:** 10.3390/biology15171515

**Published:** 2026-09-03

**Authors:** Xiuge Zhang, Tingyin Li, Cui Hua, Shanshan Jiang

**Affiliations:** 1 College of Pharmacy, Guizhou University of Traditional Chinese Medicine, Guiyang 550025, China; zxg19890113@163.com (X.Z.); 17585323864@163.com (T.L.); huacui@gzy.edu.cn (C.H.); 2 College of Basic Medicine, Guizhou University of Traditional Chinese Medicine, Guiyang 550025, China

**Keywords:** *Protegira songi*, MaxEnt, climate change, habitat suitability, pest management

## Abstract

*Protegira songi* Chen and Zhang is a monophagous defoliating pest that feeds exclusively on *Eucommia ulmoides* Oliv., an economically and medicinally important tree species widely cultivated in China. Climate change may influence the potential suitable habitat of this pest, with implications for future monitoring and management. In this study, a Maximum Entropy (MaxEnt) model integrating occurrence records and environmental variables was used to evaluate current habitat suitability and project future changes under four Shared Socioeconomic Pathway (SSP) scenarios. The minimum temperature of the coldest month (Bio6) and precipitation of the driest month (Bio14) were identified as key climatic factors associated with potential habitat suitability. Currently, suitable habitats were estimated to cover approximately 20.58% of China’s land area, with highly suitable regions mainly distributed in central and southwestern China. Future projections indicated that potential suitable habitats would undergo spatial redistribution, with core suitable areas remaining relatively stable while peripheral regions showed more variation. Under SSP245, the suitable habitat area exhibited the largest increase by the 2090s, accompanied by an overall southward shift in the centroid of suitable habitat. These findings improve understanding of climate-driven changes in the potential suitable habitat of *P. songi* and provide a scientific basis for climate-informed monitoring and adaptive pest management.

## Simple Summary

*Protegira songi* is a monophagous defoliating pest that feeds exclusively on *Eucommia ulmoides*, an economically and medicinally important tree species widely cultivated in China. Climate change may modify the potential suitable habitat of this pest, with implications for future monitoring and management strategies. In this study, we applied a MaxEnt species distribution model integrating occurrence records and environmental variables to evaluate the current and future potential suitable habitat of *P. songi* under different climate scenarios. The results indicated that climatic factors, particularly the minimum temperature of the coldest month (Bio6) and precipitation of the driest month (Bio14), were key environmental variables associated with the potential suitable habitat of *P. songi*. Currently, suitable habitats were mainly concentrated in central and southwestern China. Under future climate scenarios, core suitable areas were projected to remain relatively stable, whereas peripheral regions were projected to undergo changes in habitat suitability and spatial pattern of suitable areas. These findings provide valuable insights for climate-informed monitoring and sustainable management of *P. songi* under future climate change.

## 1. Introduction

Global climate warming is one of the most urgent environmental challenges. Intergovernmental Panel on Climate Change (IPCC) reports show that global surface temperatures are increasing and that extreme weather events, such as heatwaves and heavy rainfall, are becoming more frequent and intense [1,2,3]. Temperature and precipitation are key environmental factors that shape the development, population dynamics, and geographic distributions of herbivorous insects. Changes in these variables can alter habitat suitability, dispersal capacity, and outbreak risk, thereby shifting pest distributions and ecological impacts [4]. Insects are generally more sensitive to climate variability than vascular plants [5]. Consequently, warming has driven range shifts and expansions in many pest species, increasing the outbreak frequency and damage severity and posing serious threats to agriculture, forest health, and ecosystem security [6,7]. Understanding pest responses to climate change and potential changes in their distributions is therefore essential for designing effective monitoring and management strategies.

*Eucommia ulmoides* Oliv., the sole species in the Eucommiaceae family, is a tree of considerable economic and medicinal importance native to China. It is widely used in traditional medicine, food production, the rubber industry, ecological restoration, and animal feed development [8]. *Protegira songi* Chen et Zhang (Lepidoptera: Noctuidae) is a monophagous pest that feeds exclusively on *E. ulmoides*. In recent years, this pest has caused severe damage in major Eucommia-producing regions of China, including Guizhou, Sichuan, Hunan, Shaanxi, and Henan Provinces, and in South Korea [9,10,11,12]. Because of its high feeding capacity, strong reproductive potential, and prolonged damaging period, *P. songi* has become a major threat to the Eucommia industry. Previous studies have mainly focused on the biology, olfaction-related genes, and control strategies of *P. songi* [13,14,15,16,17,18,19,20,21]. Recently, Wu et al. applied the MaxEnt model to predict its potential distribution under future climate scenarios [22], providing an initial assessment of climate-associated habitat suitability. However, the spatial dynamics of climate-driven habitat redistribution under different climate scenarios remain insufficiently understood.

Species distribution models (SDMs) are widely used to quantify species and environment relationships and to predict potential ranges under current and future climate scenarios [23,24]. Among SDMs, the Maximum Entropy (MaxEnt) model is preferred for its strong predictive performance, robustness, and ability to generate reliable results from presence-only data and relatively small sample sizes [25,26,27]. Recently, MaxEnt has been applied to assess potential distributions and invasion risks of several agricultural and forest pests, including *Carposina coreana* [28], *Histia rhodope* [29], *Neoceratitis asiatica* [30], *Gephyraulus lycantha* [31], *Helicoverpa armigera* [32], *Amrasca biguttula* [33], and *Anoplophora chinensis* [34]. These applications have provided important support for monitoring and management programs.

In this study, we used the MaxEnt model to predict the current and future potential suitable habitat of *P. songi* in China under four Shared Socioeconomic Pathway (SSP) scenarios: SSP126, SSP245, SSP370, and SSP585. We identified the key environmental variables influencing the habitat suitability of *P. songi* and evaluated climate-driven changes in potential suitable habitat, including changes in habitat extent and spatial redistribution under future climate scenarios. The results provided a scientific foundation for the monitoring and integrated pest management of *P. songi*.

## 2. Materials and Methods

### 2.1. Collection and Screening of P. songi Occurrence Data

Occurrence records of *P. songi* were compiled from four types sources: (1) observational records from the Global Biodiversity Information Facility (GBIF, http://www.gbif.org; accessed 22 October 2025) using the keyword “*Protegira songi*”; (2) published literature records from the China National Knowledge Infrastructure (CNKI, http://www.cnki.net, accessed 22 October 2025) using the synonym “*Orthosia songi*” [35,36,37,38,39,40,41]; (3) regional monographs [42,43]; and (4) field surveys records collected in 2024–2025. All records were manually screened to remove duplicate entries at identical locations and records with incomplete or ambiguous information, yielding 54 unique occurrence sites. Each record was checked for the completeness and consistency of the geographic coordinates. For records lacking latitude and longitude, coordinates were reconstructed from provincial, county, and locality descriptions using the Baidu Map coordinate picker (http://api.map.baidu.com/lbsapi/getpoint/index.html; accessed 5 November 2025). To reduce spatial autocorrelation from clustered points, ENMTools v1.4.4 filtered the dataset to retain at most one occurrence per 5 km × 5 km grid cell. The final dataset contained 50 unique occurrence records of *P. songi*, saved as a CSV file with columns for species name, longitude, and latitude for subsequent modeling.

### 2.2. Acquisition and Evaluation of Environmental Variables

To evaluate the potential suitable habitat of *P. songi* under current and future climates, we obtained 19 bioclimatic variables from the WorldClim v2.1 database at a spatial resolution of 2.5 arc-minutes. For current conditions, we used bioclimatic data from 1970 to 2000. Future climate projections were obtained for the 2050s (2041–2060), 2070s (2061–2080), and 2090s (2081–2100) from the Beijing Climate Center Climate System Model (BCC-CSM2-MR) within CMIP6. We considered four Shared Socioeconomic Pathway (SSP) scenarios, including SSP126, SSP245, SSP370, and SSP585, which represent alternative future socioeconomic trajectories and associated greenhouse gas emission pathways. These scenarios encompass low, intermediate, medium–high, and high forcing pathways, with nominal radiative forcing levels of approximately 2.6, 4.5, 7.0, and 8.5 W m^−2^ by 2100, respectively [44,45].

To reduce model overfitting from multicollinearity among environmental predictors, we imported the 19 bioclimatic variables and occurrence records of *P. songi* into MaxEnt v3.4.4. A jackknife test was first used to evaluate the relative contribution and importance of each environmental variable to species distribution. We then used ENMTools to assess correlations among variables, conducting pairwise Pearson correlation analyses to minimize the influence of highly correlated predictors. When the absolute correlation coefficient exceeded 0.8, only the variable with the higher model contribution was retained. This procedure produced seven environmental variables for model construction: mean diurnal range (Bio2), isothermality (Bio3), temperature seasonality (Bio4), minimum temperature of the coldest month (Bio6), mean temperature of the wettest quarter (Bio8), precipitation of the wettest month (Bio13), and precipitation of the driest month (Bio14) (Table 1).

### 2.3. Model Construction and Evaluation

Occurrence records of *P. songi* and the selected environmental variables were imported into MaxEnt v3.4.4 to construct the species distribution model. The occurrence data were randomly split into training (75%) and testing (25%) sets. The threshold rule was set to “10 percentile training presence,” and the model was replicated ten times to reduce the uncertainty from random sampling. A jackknife test assessed the relative importance of environmental variables, and response curves described the relationships between those variables and habitat suitability [26,46]. The output format was set to logistic, with the maximum number of iterations set to 5000, the background points set to 10,000, and the repetition mode set to subsample [29,47].

The model performance was evaluated using the receiver operating characteristic (ROC) curve and the area under the curve (AUC). AUC values range from 0 to 1 and reflect predictive accuracy, with higher values indicating better performance. According to Wang et al. (2007) [46], AUC values below 0.5 indicate model failure, values between 0.5 and 0.7 denote acceptable performance, values between 0.7 and 0.9 indicate good performance, and values of 0.9 or higher indicate excellent predictive ability. Therefore, the AUC value served as the primary metric for assessing the model reliability and predictive performance.

### 2.4. Habitat Suitability Classification and Distribution Mapping

Habitat suitability maps generated by MaxEnt were imported into ArcGIS 10.8 for spatial analysis. Habitat suitability was classified into four categories using the Natural Breaks (Jenks) method: unsuitable (0–0.065), low suitability (0.065–0.234), moderate suitability (0.234–0.458), and high suitability (0.458–0.832) [48]. The area of each suitability class was calculated by counting raster cells to quantify the spatial extent of suitable habitats for *P. songi*.

### 2.5. Future Changes in Potential Suitable Habitat and Centroid Shifts Under Climate Scenarios

Habitat suitability maps generated by MaxEnt were exported as ASCII (ASC) files and converted into raster datasets in ArcGIS 10.8. Suitability maps were reclassified into binary rasters using a threshold value of 0.065, assigning values of 1 and 0 to suitable and unsuitable habitats, respectively. Current and future binary raster layers were then converted into polygon features using the Raster to Polygon tool. Spatial overlay analysis was performed with the Intersect tool to identify four habitat-change categories: stable, expansion, contraction, and no change. The resulting polygon layers were converted back to raster format using the Polygon to Raster tool for habitat-change analysis and area calculation.

Geographic centroids of suitable habitat patches (Value = 1) were calculated using the Mean Center tool in the ArcGIS Spatial Statistics toolbox. Centroid coordinates of the current and future suitable habitats were compared to quantify spatial displacement. The Haversine formula was used to calculate the great-circle distance between centroids, and the migration direction was inferred from changes in centroid latitude and longitude.

## 3. Results

### 3.1. Model Predictive Performance

**Figure 1:**
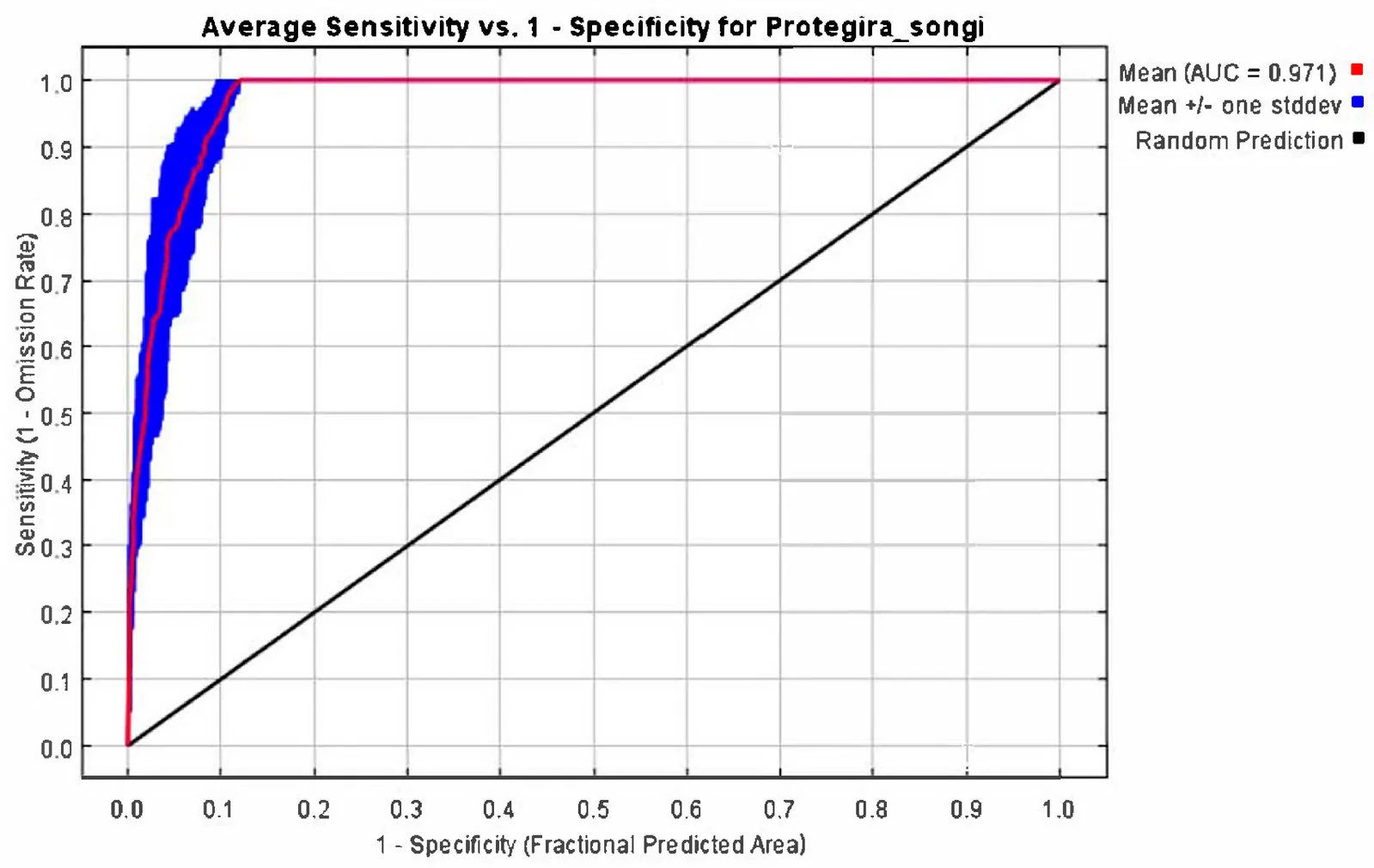
ROC curve and AUC value generated by MaxEnt model.

The predictive performance of the MaxEnt model was assessed using the receiver operating characteristic (ROC) curve and the corresponding area under the curve (AUC). The mean AUC value was 0.971, indicating excellent predictive performance according to the established evaluation criteria. This result demonstrates that the MaxEnt model accurately captured the relationships between species occurrence records and environmental variables and enabled reliable predictions of the potential suitable habitat of *P. songi* in China (Figure 1).

### 3.2. Analysis of Key Environmental Variables

**Figure 2:**
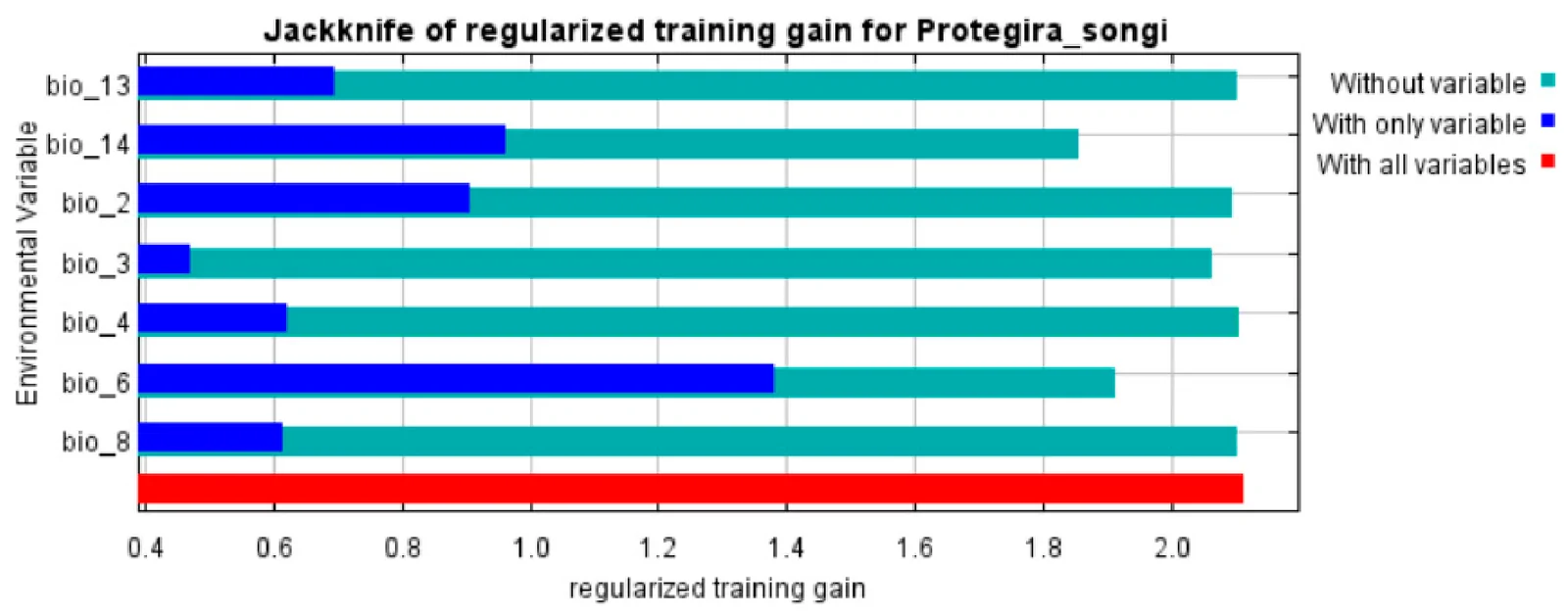
Jackknife analysis of the importance of seven environmental variables to the predicted habitat suitability of *P. songi*.

The percent contribution, permutation importance, and jackknife analyses consistently identified a core set of bioclimatic variables as the primary predictors of habitat suitability for *P. songi* (Table 1; Figure 2). The minimum temperature of the coldest month (Bio6), precipitation of the driest month (Bio14), isothermality (Bio3), temperature seasonality (Bio4), mean diurnal temperature range (Bio2), and precipitation of the wettest month (Bio13) together explained most of the model’s predictive power. Bio6 showed the highest contribution and permutation importance across all evaluation metrics.

**Figure 3:**
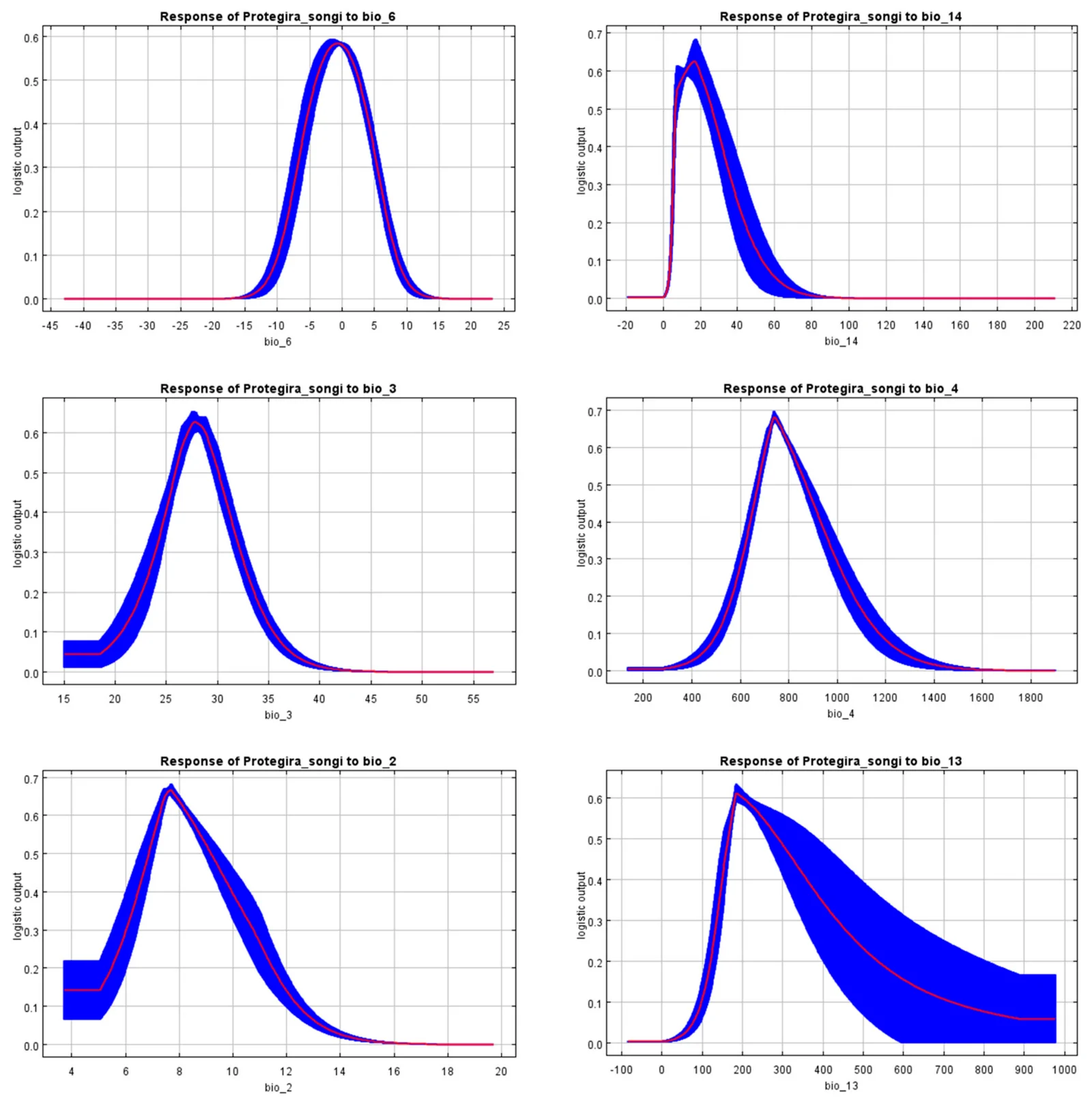
Response curves of key environmental variables contributing to the predicted habitat suitability of *P. songi*. The x-axis represents environmental variables, and the y-axis represents predicted suitability (logistic output). Curves show the mean response across 10 MaxEnt replicate runs.

Jackknife analyses indicated that Bio6, Bio14, Bio3, and Bio2 produced the largest gains when used alone and that removing them caused the largest declines in model performance, highlighting their strong independent informational value. Response curve analysis quantified the environmental tolerance ranges of *P. songi* as follows: Bio6 ranged from −4.01 to 2.56 °C, Bio14 from 6.60 to 26.42 mm, Bio3 from 25.87 to 30.12, Bio4 from 671.33 to 872.64, Bio2 from 6.86 to 9.20 °C, and Bio13 from 164.25 to 289.71 mm (Figure 3; Table 2).

### 3.3. Predicted Suitable Habitat of P. songi Under Current Climatic Conditions

**Figure 4:**
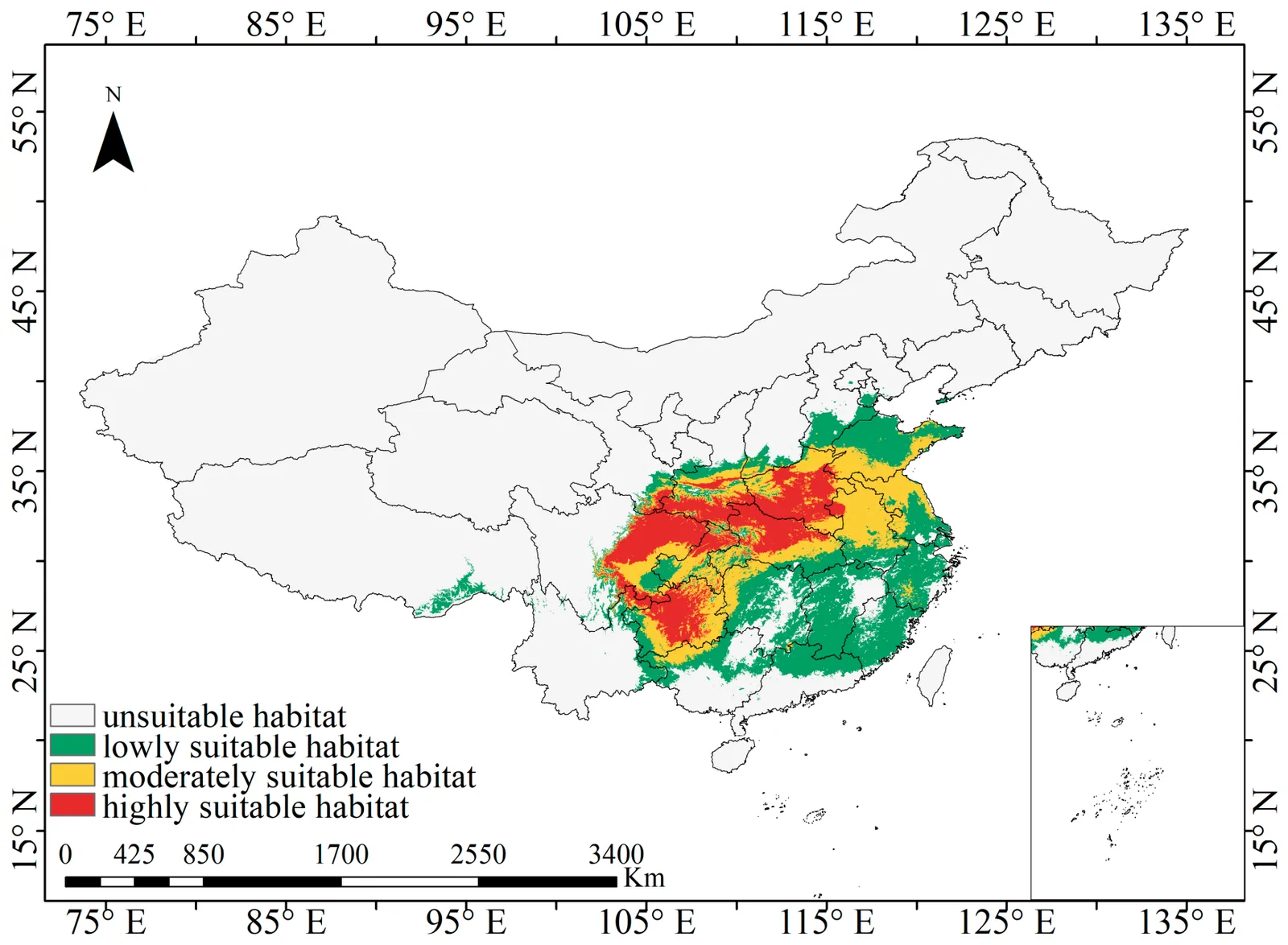
Potential suitable habitat of *P. songi* in China under current climate conditions.

Under current climatic conditions, the potential suitable habitats of *P. songi* were mainly located in central, southwestern, and parts of eastern China, forming a relatively continuous distribution belt extending from southern Shaanxi through western Hubei and Chongqing to eastern Sichuan and northern Guizhou (Figure 4). The total potential suitable area was estimated at 1,975,600 km^2^, representing approximately 20.58% of China’s land area (Table 3).

Highly suitable habitats covered 439,200 km^2^ (4.58% of China’s land area) and formed a relatively continuous core suitable area centered on central Henan, southern Shaanxi, western Hubei, Chongqing, eastern Sichuan, and northern Guizhou. Moderately suitable habitats occupied 594,500 km^2^ (6.19%) and were mainly located around the highly suitable areas, extending across northern and southern Henan, southwestern Shandong, northern Anhui, northwestern Jiangsu, southern Zhejiang, most of Hubei, northern Hunan, large parts of Sichuan, and much of Guizhou. Low-suitability habitats covered 941,900 km^2^ (9.81%) and occurred mainly in the peripheral areas surrounding the moderately suitable habitats, including southern Hebei, northwestern Shandong, Fujian, Guangdong, Guangxi, southern Jiangxi, eastern Yunnan, and small areas of southeastern Tibet. The remaining 79.42% of China’s land area was classified as unsuitable. The habitat suitability showed a gradual decline from the core suitable areas toward surrounding regions (Figure 4, Table 3).

### 3.4. Predicted Suitable Habitat of P. songi Under Future Climate Scenarios

**Figure 5:**
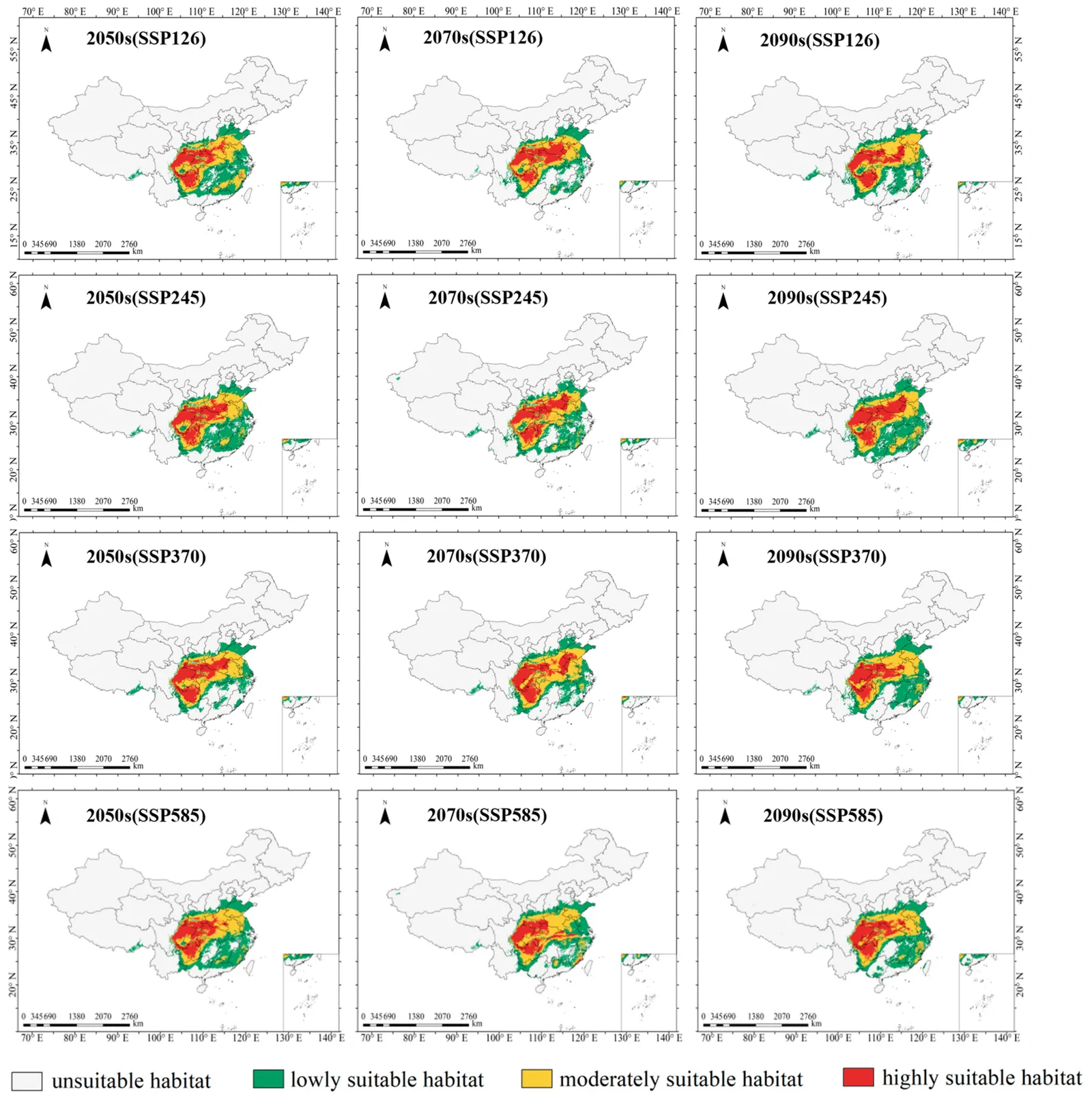
Potential suitable habitat of *P. songi* in China under future climatic scenarios.

Under future climate scenarios, suitable habitats for *P. songi* were projected to remain mainly concentrated in central and southwestern China, showing a similar spatial pattern to the current potential suitable habitats (Figure 5). However, the extent of suitable habitats and the proportions of different suitability classes varied among SSP scenarios and projection periods (Table 3). In the 2050s, the total suitable habitat area increased under SSP126, SSP245, and SSP585, reaching 202.63 × 10^4^, 199.93 × 10^4^, and 202.69 × 10^4^ km^2^, respectively, whereas SSP370 showed a decrease to 177.84 × 10^4^ km^2^. By the 2070s, the total suitable habitat area declined under most scenarios, with SSP126 showing the smallest extent (174.72 × 10^4^ km^2^; −11.56%), while SSP370 and SSP585 maintained relatively larger suitable habitat areas. In the 2090s, SSP245 exhibited the largest expansion in suitable habitat extent, reaching 223.82 × 10^4^ km^2^ (+13.29%), accompanied by increases in highly, moderately, and lowly suitable habitats. In contrast, SSP126 showed a slight decrease (−3.68%), whereas SSP370 and SSP585 exhibited only minor changes. Across suitability classes, low-suitability areas declined under all scenarios in the 2050s and 2070s, with the largest reduction observed under SSP370 in the 2050s (−27.24%). This decreasing trend continued under SSP126, SSP370, and SSP585 in the 2090s, whereas SSP245 showed an increase (+10.73%). Moderately suitable habitats expanded consistently under SSP370 and SSP585 throughout the three projection periods, although the largest increase was observed under SSP126 in the 2050s (+17.07%). Highly suitable habitats showed a consistent increasing trend under SSP245, reaching the highest expansion (+22.75%) in the 2090s.

### 3.5. Spatial Changes in Potential Suitable Habitat Under Future Climate Scenarios

**Figure 6:**
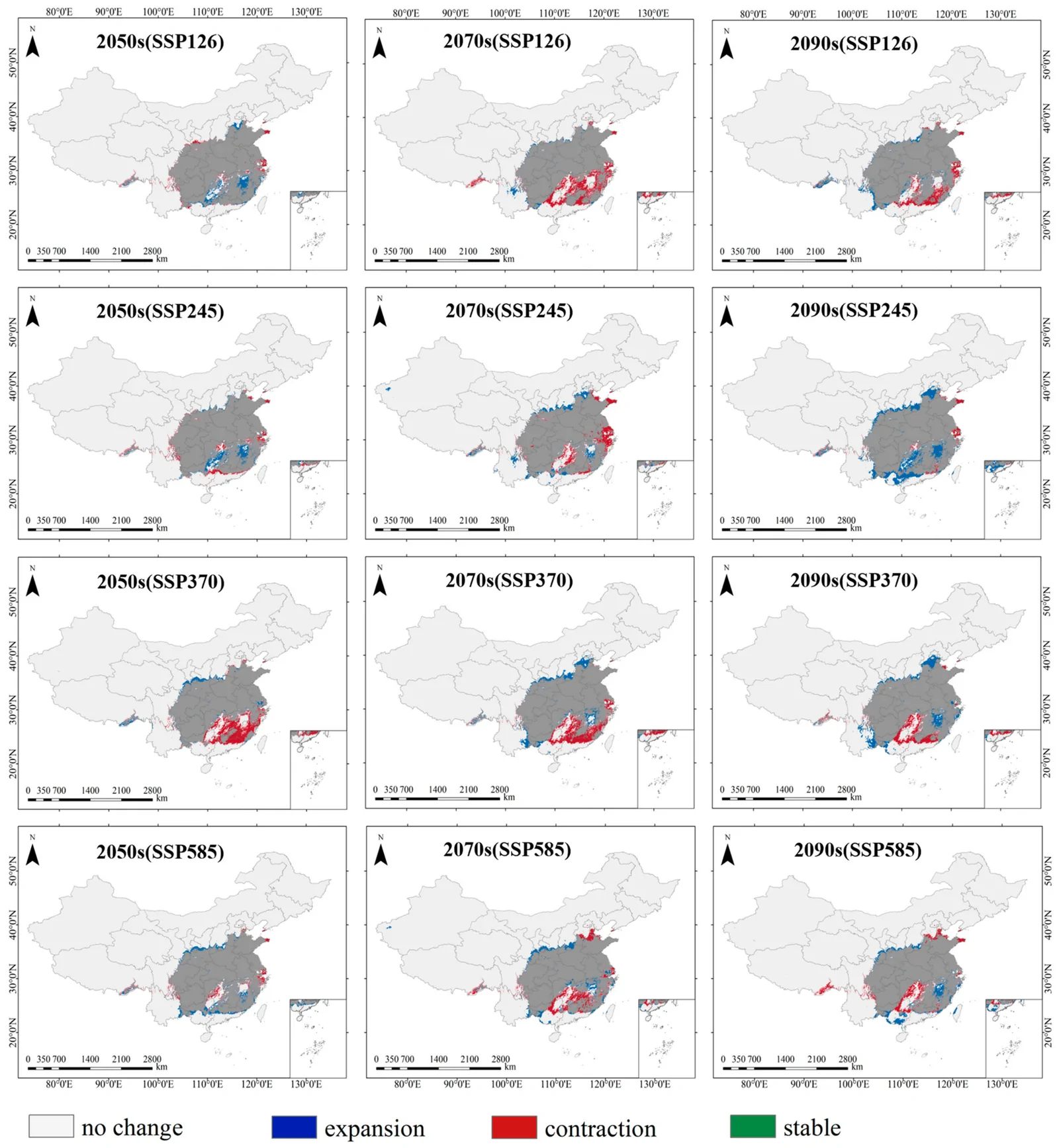
Spatial changes in potential suitable habitat of *P. songi* under future climate scenarios.

Under future climate scenarios, the potential suitable habitat of *P. songi* remained largely stable, with localized expansion and contraction occurring mainly along the margins of the current suitable areas (Figure 6, Table 4). By the 2050s, the habitat changes were relatively limited across SSP scenarios. Stable suitable habitat areas under SSP126, SSP245, and SSP585 were 190.50 × 10^4^, 188.22 × 10^4^, and 190.45 × 10^4^ km^2^, respectively. SSP370 showed a smaller stable area (170.54 × 10^4^ km^2^) and a larger contraction area (27.16 × 10^4^ km^2^). Expansion areas under SSP126, SSP245, and SSP585 were similar (12.17–12.64 × 10^4^ km^2^), whereas SSP370 had the lowest expansion (7.40 × 10^4^ km^2^). By the 2070s, differences between the scenarios became more pronounced. SSP126 showed a decline in stable suitable habitat area (169.94 × 10^4^ km^2^) and increased contraction (27.76 × 10^4^ km^2^), whereas SSP245, SSP370, and SSP585 showed larger expansion (14.04–15.68 × 10^4^ km^2^). By the 2090s, SSP245 showed the largest expansion (32.76 × 10^4^ km^2^, 3.41%), accompanied by increased stable suitable habitat (191.61 × 10^4^ km^2^) and reduced contraction (6.08 × 10^4^ km^2^). In contrast, SSP126, SSP370, and SSP585 exhibited relatively larger contractions, with contracted areas of 17.21 × 10^4^, 16.06 × 10^4^, and 17.07 × 10^4^ km^2^, respectively. Spatially, newly suitable areas were mainly located along the northern and western margins, whereas contracted areas were concentrated in the southern and southeastern regions.

### 3.6. Centroid Shifts of Potential Suitable Habitat Under Future Climate Scenarios

**Figure 7:**
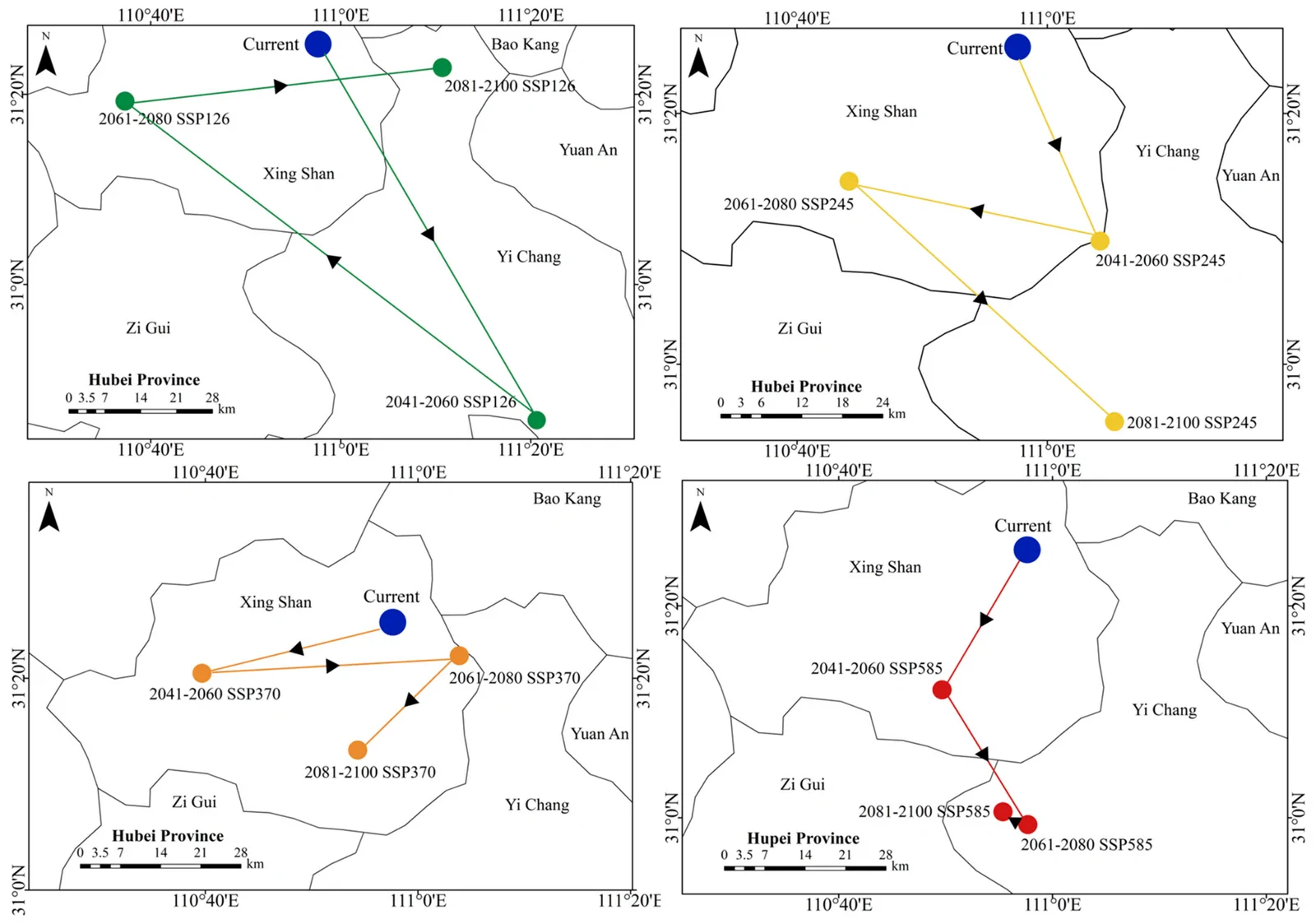
Centroid shifts of potential suitable habitat of *P. songi* under future climate scenarios. Different colors represent different SSP scenarios, and dots indicate centroid locations across different projection periods.

Under current climatic conditions, the centroid of the potential suitable habitat of *P. songi* is located in northern Xingshan County, Yichang City, Hubei Province (110.9605° E, 31.4216° N) (Figure 7). Future climate projections indicated distinct centroid shift patterns across Shared Socioeconomic Pathways (SSPs), generally showing a southward tendency but differing in distance and trajectory (Figure 7, Table 5). Under SSP126, the centroid shifted 81.30 km southward to southern Yiling District (111.3438° E, 30.7620° N) by the 2050s, followed by a northwestward shift of 34.04 km in the 2070s, and a southeastward shift of 21.17 km in the 2090s. Under SSP245, the centroid showed an overall southward displacement, reaching 111.0704° E, 31.1636° N in the 2050s, 110.7359° E, 31.2431° N in the 2070s, and 111.0897° E, 30.9237° N in the 2090s, with a maximum displacement of 56.69 km by the 2090s. In contrast, under SSP370, the centroid remained relatively close to its current location, shifting 29.74 km southwestward, 11.49 km southeastward, and 23.00 km southward in the 2050s, 2070s, and 2090s, respectively. Under SSP585, the centroid generally shifted southward, with displacement distances of 27.54 km, 48.05 km, and 45.95 km in the 2050s, 2070s, and 2090s, respectively.

## 4. Discussion

Climate change has altered environmental conditions that influence pest distribution patterns and has increased the need to assess potential shifts in habitat suitability under future climate scenarios [49]. Species distribution models (SDMs) have therefore become valuable tools for evaluating habitat suitability and supporting risk assessment and adaptive management of forest pests [50]. In this study, the MaxEnt model was applied to estimate the current and future potential suitable habitats of *P. songi* in China under multiple climate scenarios. The model showed excellent predictive performance, with an AUC of 0.971, indicating high accuracy in predicting *P. songi*’s habitat suitability. The results provide a basis for interpreting the climatic drivers and future changes in the potential suitable habitat of *P. songi*.

### 4.1. Key Environmental Factors Associated with the Potential Suitable Habitat of P. songi

Analysis of environmental variables indicated that temperature and precipitation were the major climatic factors associated with the potential suitable habitat of *P. songi*. Among these, the minimum temperature of the coldest month (Bio6) and precipitation of the driest month (Bio14) showed the highest contributions based on permutation importance analysis. These findings are consistent with Wu et al. (2025) [22], who also identified temperature-related variables and seasonal precipitation as the primary factors influencing the predicted habitat suitability of *P. songi*. Overall, temperature-related variables contributed more than precipitation variables, highlighting the predominant role of thermal conditions in shaping the potential suitable habitat of this species.

The importance of Bio6 highlights the ecological role of winter temperature in constraining the potential suitable habitat of temperate and subtropical insects, as overwinter survival strongly influences the population persistence and range boundaries [4,51]. *P. songi* overwinters as pupae in shallow soil layers and has limited cold tolerance [13]. In this study, suitable habitats were associated with a coldest-month temperature range of −4.01 to 2.56 °C, suggesting that low winter temperatures may constrain overwintering suitability and restrict potential range expansion. Precipitation, particularly during dry periods, may also influence the habitat suitability by regulating soil moisture conditions. Previous studies have shown that soil moisture affects pupal development and adult emergence in soil-dwelling insects [52], suggesting that precipitation may indirectly affect the population persistence of *P. songi* by modifying the developmental environment of overwintering pupae. The contributions of Bio2, Bio3, and Bio4 further suggest that *P. songi* responds to both absolute temperature and temperature variability. Together, temperature and precipitation contribute to the potential suitable habitat of *P. songi*, a pattern consistent with that reported for other subtropical Lepidopteran pests [53,54]. In addition to climate, host plant distribution may further influence pest occurrence [55]. Bio6 and Bio14 are also key climatic variables affecting the potential suitable habitat of *E. ulmoides* [56], while temperature seasonality and precipitation contribute substantially to its habitat suitability [57,58]. The similarity in climatic determinants of *P. songi* and its host suggests that suitable climatic conditions and host availability may contribute to the persistence of *P. songi* populations in climatically suitable areas.

### 4.2. Current Potential Suitable Habitat of P. songi

Under current climatic conditions, the potential suitable habitat of *P. songi* was characterized by a relatively continuous high-suitability belt extending across central China, with core suitable areas located in central Henan, southern Shaanxi, western Hubei, Chongqing, eastern Sichuan, and northern Guizhou. These regions broadly correspond to the major distribution range of *E. ulmoides*, suggesting that the current distribution pattern of *P. songi* may be associated with the combined effects of climatic suitability and host availability. Similar patterns have been reported for other herbivorous insects, such as *Phthorimaea absoluta* and *Spodoptera frugiperda*, in which suitable climatic conditions provide favorable conditions for population persistence, while host availability further influences the realized distribution and population establishment [59,60]. Field surveys have also reported higher pest densities and more severe damage in highly suitable areas [15], supporting the spatial consistency of the model predictions.

Furthermore, the habitat suitability gradually declined from the highly suitable areas to the surrounding moderately and lowly suitable areas, and the predicted suitability pattern showed broad-scale spatial correspondence with the distribution of host plantations. This pattern suggests that areas with abundant host resources may represent potential areas for enhanced monitoring. In contrast, the northwest, northeast, and the Qinghai–Tibet Plateau remained largely unsuitable because of unfavorable climatic conditions and limited host availability.

### 4.3. Future Changes in Potential Suitable Habitat of P. songi Under Climate Change Scenarios

Simulations under multiple Shared Socioeconomic Pathways (SSPs) indicated that future climate change was projected to largely maintain the current spatial pattern of potential suitable habitat of *P. songi* in China while resulting in gradual changes in habitat suitability and spatial redistribution. Highly suitable habitats were projected to remain concentrated in the current core regions, particularly in southwestern and central–southern China, whereas most habitat changes were expected to occur along the range margins. This “stable core–dynamic margin” pattern, also reported for other forest pests under climate-change scenarios [61,62], suggests that future range shifts are more likely to occur through peripheral expansion and contraction rather than a large-scale displacement of the overall potential suitable habitat. The magnitude and direction of habitat changes varied among SSP scenarios. By the 2090s, SSP245 showed the largest expansion in suitable habitat extent, which may be associated with an increased climatic suitability at habitat margins under moderate warming conditions without excessive climatic constraints. In comparison, SSP126, representing a low forcing pathway under stronger climate mitigation efforts, showed relatively minor changes in suitable habitat extent by the 2090s, suggesting that reduced climatic pressure may constrain large-scale shifts in the potential suitable habitats of *P. songi*. Under higher forcing scenarios (SSP370 and SSP585), however, habitat responses became more complex, with decreased suitability in some currently suitable regions accompanied by the emergence of newly suitable habitats elsewhere. Similar nonlinear responses to warming have been observed in ectothermic organisms, where moderate warming can enhance biological performance, whereas excessive warming may exceed the thermal limits and reduce population performance [63,64]. The projected expansion of moderately and highly suitable habitats under several scenarios indicated an increased potential for population establishment and persistence. For *P. songi*, which exhibits overlapping generations [11], improved climatic suitability may accelerate development and shorten generation intervals, potentially increasing population growth potential. However, outbreak risks are also regulated by host availability, natural enemies, and local ecological conditions [65,66]. Therefore, regions undergoing transitions toward higher suitability should be prioritized for long-term monitoring and early-warning programs.

The centroid shift analysis further revealed scenario-specific spatial responses of the potential suitable habitat of *P. songi* under future climate scenarios. The current centroid of potential suitable habitats, located in northern Xingshan County, Hubei Province, showed an overall southward tendency, although the migration trajectories varied among SSP scenarios. SSP245 exhibited the strongest long-term southward displacement (56.69 km by the 2090s), whereas SSP370 showed relatively limited movement. This pattern was consistent with previous projections for *P. songi* [22] but differed from the poleward or elevational shifts commonly reported for many lepidopteran pests under climate warming [67]. These differences suggest that future patterns of potential suitable habitat may reflect the combined effects of climatic conditions and host availability. The spatial consistency between highly suitable habitats projected for *P. songi* and the distribution of its host plant *E. ulmoides* further suggests that host availability may influence the potential establishment of populations under future climate scenarios [56]. Consequently, southwestern and central–southern China may remain important regions for monitoring potential habitat changes, while newly suitable peripheral areas warrant further attention for evaluating possible range expansion under future climate scenarios.

### 4.4. Limitations and Future Perspectives

This study evaluated the climate-driven potential suitable habitat of *P. songi*, while recognizing that its occurrence and establishment are influenced by multiple ecological processes. As a monophagous herbivorous insect, *P. songi* is strongly dependent on its host plant, and future incorporation of host distribution and other biotic factors, such as natural enemy pressure, would provide further insights into the ecological factors shaping its habitat suitability. In addition, species distribution models simplify complex ecological systems and may not fully represent interactions among climatic and biological factors. Although spatial thinning was applied to reduce the clustering effects, residual spatial autocorrelation may still influence model evaluation when occurrence records are randomly partitioned. The suitability classes derived from MaxEnt represent relative climatic suitability and should therefore be interpreted as indicators of potential habitat suitability rather than direct estimates of pest abundance or damage severity without independent field validation. Furthermore, future projections were based on a single CMIP6 GCM (BCC-CSM2-MR), and uncertainties associated with climate model selection may influence the magnitude of projected habitat suitability changes and centroid shifts. Future studies integrating improved model calibration, spatially independent validation, multi-GCM ensembles, and additional ecological information will further enhance the robustness and interpretation of climate-driven habitat suitability assessments for *P. songi*.

## 5. Conclusions

This study applied the MaxEnt model to assess the current and future potential suitable habitat of *P. songi* in China under climate change scenarios. The results showed that current potential suitable habitats were mainly concentrated in central and southwestern China, and these regions were projected to remain important under future scenarios, with spatial redistribution rather than a complete displacement of the current suitable habitat pattern. The minimum temperature of the coldest month (Bio6) and the precipitation of the driest month (Bio14) were identified as key climatic variables affecting the potential suitable habitat of *P. songi*. Among the four SSP scenarios (SSP126, SSP245, SSP370, and SSP585), SSP245 showed the highest expansion in suitable habitat extent by the 2090s, accompanied by an overall southward centroid shift and scenario-specific migration trajectories. These findings highlight the importance of considering future climate projections together with host-plant information to better understand potential habitat changes and provide a scientific basis for climate-adaptive monitoring, early warning, and integrated management of *P. songi*.

## Figures and Tables

**Table 1:** Contribution rates of environmental variables in constructing the MaxEnt model.

Variables	Description	Unit	Percent Contribution (%)	Permutation Importance (%)
Bio6	minimum temperature of the coldest month	°C	42.4	81.7
Bio14	precipitation of the driest month	mm	41.6	6
Bio3	isothermality (bio 2/bio 7) × 100		8.7	8.1
Bio4	temperature seasonality (standard deviation × 100)		5.3	1.9
Bio2	mean diurnal range	°C	1.1	0.5
Bio8	mean temperature of the wettest quarter	°C	0.8	1.6
Bio13	precipitation of the wettest month	mm	0.2	0.3

**Table 2:** Potential ranges of suitable environmental variables for *P. songi*.

Environmental Variables	Suitable Range	Optimal Values
Bio6	−4.01–2.56 °C	−0.69 °C
Bio14	6.60–26.42 mm	16.74 mm
Bio3	25.87–30.12	27.85
Bio4	671.33–872.64	741.96
Bio2	6.86–9.20 °C	7.68 °C
Bio13	164.25–289.71 mm	184.45 mm

**Table 3:** Predicted suitable habitat areas for *P. songi* in China under current and future climatic scenarios.

Habitat Suitability Class	Current Area	Projection Period	SSP126	SSP245	SSP370	SSP585
Highly suitable habitat	43.92	2050s	40.48 (−7.83%)	48.75 (+11.00%)	48.39 (+10.18%)	43.50 (−0.96%)
		2070s	45.79 (+4.26%)	47.56 (+8.29%)	48.09 (+9.49%)	39.84 (−9.29%)
		2090s	42.09 (−4.17%)	53.91 (+22.75%)	43.62 (−0.68%)	46.70 (+6.33%)
Moderately suitable habitat	59.45	2050s	69.60 (+17.07%)	62.01 (+4.31%)	60.92 (+2.47%)	65.86 (+10.78%)
		2070s	56.42 (−5.10%)	57.56 (−3.18%)	66.20 (+11.35%)	68.99 (+16.05%)
		2090s	67.29 (+13.19%)	65.61 (+10.36%)	64.44 (+8.39%)	61.26 (+3.04%)
Lowly suitable habitat	94.19	2050s	92.55 (−1.74%)	89.17 (−5.33%)	68.53 (−27.24%)	93.33 (−0.91%)
		2070s	72.51 (−23.02%)	87.88 (−6.70%)	75.19 (−20.17%)	82.88 (−12.01%)
		2090s	80.91 (−14.10%)	104.30 (+10.73%)	93.06 (−1.20%)	87.67 (−6.92%)
Total suitable habitat	197.56	2050s	202.63 (+2.57%)	199.93 (+1.20%)	177.84 (−9.98%)	202.69 (+2.60%)
		2070s	174.72 (−11.56%)	193.00 (−2.31%)	189.48 (−4.09%)	191.71 (−2.96%)
		2090s	190.29 (−3.68%)	223.82 (+13.29%)	201.12 (+1.80%)	195.63 (−0.98%)

Note: Values in the “Current area” column represent potential suitable habitat areas under current climatic conditions (×10^4^ km^2^). For each SSP scenario, values represent potential suitable habitat areas (×10^4^ km^2^), with percentage changes relative to the corresponding current values shown in parentheses. The proportions of potential suitable habitat area were calculated relative to the total land area of China (~9.60 × 10^6^ km^2^).

**Table 4:** Spatial changes in potential suitable habitat of *P. songi* in China under future climate scenarios.

Stage	Scenarios	Area (×10^4^ km^2^)			Change (%)		
		Expansion Area	Stable Area	Reduced Area	Expansion Rate	Stability Rate	Reduction Rate
2050s	SSP126	12.56	190.50	7.20	1.31	19.84	0.75
	SSP245	12.17	188.22	9.48	1.27	19.61	0.99
	SSP370	7.40	170.54	27.16	0.77	17.76	2.83
	SSP585	12.64	190.45	7.25	1.32	19.84	0.75
2070s	SSP126	4.96	169.94	27.76	0.52	17.70	2.89
	SSP245	14.04	179.55	18.14	1.46	18.70	1.89
	SSP370	15.68	173.96	23.73	1.63	18.12	2.47
	SSP585	14.18	177.75	19.95	1.48	18.51	2.08
2090s	SSP126	10.00	180.49	17.21	1.04	18.80	1.79
	SSP245	32.76	191.61	6.08	3.41	19.96	0.63
	SSP370	19.62	181.64	16.06	2.04	18.92	1.67
	SSP585	15.22	180.64	17.07	1.59	18.82	1.78

**Table 5:** Centroid locations and displacement distances of potential suitable habitats of *P. songi* in China under future climate scenarios.

Stage	Scenarios	Centroid Coordinate		Displacement Distance (km)
		Longitude (° E)	Latitude (° N)	
current		110.9605	31.4216	0.0000
2050s	SSP126	111.3438	30.7620	81.3008
	SSP245	111.0704	31.1636	30.5300
	SSP370	110.6614	31.3412	29.7400
	SSP585	110.8277	31.2013	27.5400
2070s	SSP126	110.6217	31.3215	34.0400
	SSP245	110.7359	31.2431	29.1300
	SSP370	111.0648	31.3689	11.4900
	SSP585	110.9616	30.9894	48.0500
2090s	SSP126	111.1784	31.3803	21.1700
	SSP245	111.0897	30.9237	56.6900
	SSP370	110.9055	31.2201	23.0000
	SSP585	110.9228	31.0095	45.9500

## Data Availability

The raw data supporting the conclusions of this article will be made available by the authors on request.
